# Assessment of Obstructive Sleep Apnoea and Sleeping Laterality by Evaluating Upper Eyelid Distraction: A Prospective, Comparative Polysomnographic Study

**DOI:** 10.7759/cureus.9566

**Published:** 2020-08-05

**Authors:** Ru Jin Eugene Ting, Nandini Singh, Melvin Ling, Sascha K Spencer, Muhammad A Khan, Anup Desai, Ashish Agar, Ian C Francis

**Affiliations:** 1 Ophthalmology, The University of Sydney, Sydney, AUS; 2 Ophthalmology, Sydney Hospital and Sydney Eye Hospital, Sydney, AUS; 3 Ophthalmology, The Prince of Wales Hospital, Sydney, AUS; 4 Ophthalmology, University of New South Wales, Sydney, AUS; 5 Respiratory Medicine, The Prince of Wales Hospital, Sydney, AUS

**Keywords:** upper eyelid distraction assessment, obstructive sleep apnoea, polysomnography, floppy eyelid syndrome, lateralising eyelid sleep compression study

## Abstract

Objective

Our goal was to evaluate upper eyelid laxity by digital distraction, with the aim to determine sleeping laterality and the likelihood of obstructive sleep apnoea (OSA), and correlate these findings with polysomnography (PSG).

Design

We conducted a prospective, single-centre multidisciplinary study in a large sleep and respiratory department and an ophthalmology department within a tertiary referral university teaching hospital.

Methods

Patients with known OSA were evaluated using techniques based on the Lateralising Eyelid Sleep Compression (LESC) study. Upper eyelid laxity was assessed by two masked investigators, and the eyelid side with greater laxity was regarded as indicative of that patient’s sleeping laterality: ‘investigator-detected sleeping laterality’ (ID SL). Each patient was then asked about the laterality of his or her accustomed sleeping position: ‘patient-reported sleeping laterality’ (PR SL). PSG was conducted according to the standard protocol of the Department of Sleep and Respiratory Medicine (DSRM). ‘Polysomnography-detected sleeping laterality’ (PSG SL) permitted the extraction of sleep positional data by two masked sleep scientists.

Results

The reliability of the LESC technique for diagnosing ID SL was demonstrated to be statistically significant (p<0.01). Upper eyelid laxity was significantly greater on the patients’ sleeping side (t=6.340, df=45, p<0.01, two-tailed). There was a significant correlation between PR SL and ID SL (r_s _=0.33). However, PSG SL did not correlate with sleeping laterality compared with both ID SL and PR SL.

Conclusion

This study confirms that there is a statistically significant correlation of sleeping laterality with increasing upper eyelid laxity in OSA. Counterintuitively, PSG SL correlated poorly with ID SL and PR SL. This may likely be explained by the technical limitations implicit in current PSG techniques.

## Introduction

Floppy eyelid syndrome (FES) is a condition characterised by a totally evertible upper eyelid and a pliable tarsal plate, with inflamed conjunctiva. FES may result in ocular discomfort, blurred vision, and papillary conjunctivitis [1,2]. It is frequently associated with obstructive sleep apnoea (OSA), which has been reported to be observed in up to 85% of the FES population [2].

Polysomnography (PSG) is the gold-standard method for OSA diagnosis, but its use as an accessory tool in the diagnosis of FES has not previously been studied in the literature [3.4]. The current masked, prospective study compared sleeping laterality in individuals with definite or highly suspected OSA by three methods. These were ‘investigator-detected sleeping laterality’ (ID SL), ‘patient-reported sleeping laterality’ (PR SL), and ‘polysomnography-detected sleeping laterality’ (PSG SL). This study is the first to compare PSG SL with PR SL and ID SL. It was postulated that in patients with suspected OSA, greater upper eyelid laxity would be observed on the sleeping side, confirming the findings of the Upper Eyelid Distraction Assessment (UEDA), performed by our group in 2014 [3].

In the current study, the authors hypothesised that ID SL as demonstrated by the UEDA would correlate positively with the patients’ PSG SL. This should assist not only in diagnosing OSA, allowing for potential management of OSA, but also the patient’s sleeping laterality, and its multiple systemic and ophthalmological associations including FES.

## Materials and methods

A prospective, multidisciplinary, masked, single-centre study was conducted in the Department of Sleep and Respiratory Medicine (DSRM) and the Department of Ophthalmology. These departments were within a large, tertiary referral university teaching hospital (The Prince of Wales Hospital) in Sydney, Australia, and the University of New South Wales, Sydney, Australia. Ethics approval was obtained from the Prince of Wales Hospital Human Research and Ethics Committee. The study was carried out in adherence to the tenets of the Declaration of Helsinki.

Recruitment

Patients undergoing PSG for highly suspected or known OSA at the sleep laboratory of the DSRM were recruited over a 16-week period. Patients with a history of facial nerve palsy, eyelid trauma or surgery, and patients <18 years of age were excluded. All patients gave informed written consent.

Eyelid examination

A thorough explanation as to the method of UEDA was provided to each patient. The details of this examination have been previously presented and reported by Figueira et al. (a study of which the current authors were also co-authors). They are summarised in the following paragraph [3].

**Figure 1:**
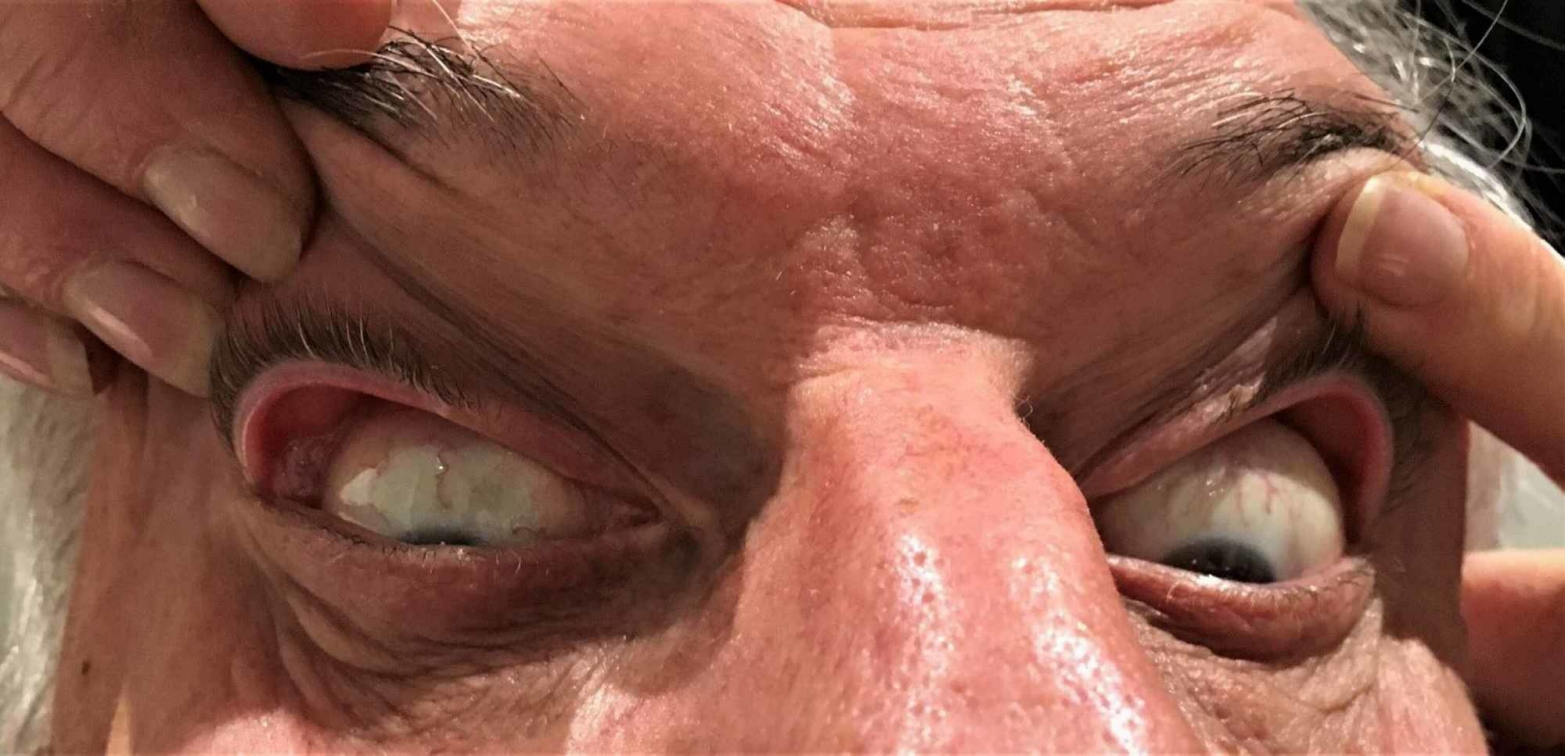
Evaluation of lid laxity For the purposes of the photograph, the ophthalmic assistant is using her fingers and thumbs on the patient’s upper eyelids laterally, gently distracting them superiorly and anterolaterally to demonstrate asymmetric lid laxity in this case. Here, the right upper lid is more distractible than the left

**Figure 2:**
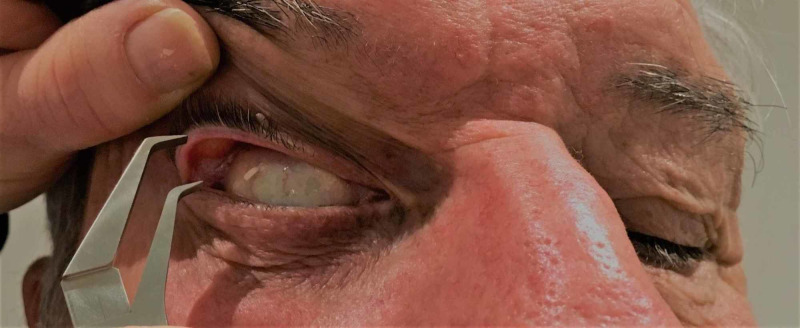
Assessment of upper eyelid distraction distance Upper eyelid distraction distance, measured from the posterior margin of the upper lid to the bulbar conjunctiva, is assessed using sterile calipers. This procedure is then repeated by the co-investigator. At the conclusion of the two assessments, the patient is asked to report their accustomed sleeping side. This is reported as PR SL. To minimise bias, the ID SL result is not divulged to the patient PR SL: patient-reported sleeping laterality; ID SL: investigator-detected sleeping laterality

Following an explanation of the proposed technique, the patient is seated immediately in front of the investigator, who has clean, dry hands. The patient is asked to look downwards and to elevate his or her chin slightly. The eyelids are gently cleaned of excess oil laterally using a facial tissue. The investigator carefully places his/her thumbs, bilaterally and simultaneously, on the lateral thirds of the upper eyelids of the patient, close to the lid margin. Simultaneously, the investigator distracts both upper eyelids superiorly and anterolaterally (Figure 1). The upper eyelid distraction distance is measured by the investigator from the posterior margin of the upper lid to the bulbar conjunctiva using sterile 0.05-mm graded calipers (Figure 2). The side with greater laxity is labelled as ID SL. The result of the UEDA is not revealed to the patient. Each patient is then asked about the laterality of his or her normal sleeping position (PR SL).

Polysomnography assessment

Overnight PSG was performed using a Philips Respironics Alice system (Philips Respironics, Murrysville, PA), powered by Sleepware G3 (Philips Respironics, Murrysville, PA) sleep diagnostic software. This included measurements of body position assumed during sleep. The data collected included electroencephalogram signals, bilateral electrooculogram, submental electromyography, electrocardiography, bilateral anterior tibial muscle activity, arterial oxygen saturation, sound, respiratory thoracoabdominal movements, airflow (nasal pressure and oronasal thermocouples), and body position.

Body position was determined by positional sensors (Synergy Protech, Woodinville, WA), which were attached to the midline of the abdominal wall. The sensors differentiated between upright, left, right, prone, and supine positions. All signals were recorded with digital sampling, filtering, storage and recording technology.

The data were interpreted by two sleep scientists from DSRM, masked to any other information obtained in this study. Using these data, the sleeping position most assumed overnight was determined and was described as PSG SL.

Statistical analysis

Statistical analysis was performed using IBM SPSS Statistics for Windows, version 22.0 (IBM Corp., Armonk, NY). Normality was assessed using the Shapiro-Wilk test. Values for reported parameters are presented as either median [interquartile range (IQR)] or mean ±standard deviation. Spearman’s rho (ρ) and Pearson’s correlation analysis were performed to assess the variability of investigator findings. A paired t­-test analysis was used to assess the difference in upper eyelid laxity between the sleeping and non-sleeping sides. Spearman’s ρ correlation analysis was used to assess the relationship between upper eyelid laxity and the sleeping side. A p-value of <0.05 was considered statistically significant.

## Results

Twenty-eight patients were recruited from the DSRM, Prince of Wales Hospital in Sydney, Australia. The median age of the patients was 58 years (range: 42.25-73.75 years), and 71% of the cohort was male.

The upper eyelid laxity examination was assessed for reliability using Spearman’s ρ and Pearson’s two-tailed correlation analysis. The former was used to assess ID SL, while the latter was used to assess investigator distraction distance measurements for both right and left upper eyelids. UEDA was found to be correlated with the distraction distance at a statistically significant level (p: 0.01) (Table 1).

**Table 1:** Statistical analysis of UEDA and sleeping laterality ^*^Significant positive associations for distraction distance between investigator findings UEDA: Upper Eyelid Distraction Assessment

	r/r_s*_	N	P-value
Investigator-detected laterality	0.588	28	0.01
Right upper lid distraction distance	0.734	28	<0.01
Left upper lid distraction distance	0.726	28	<0.01

Upper eyelid laxities of the sleeping and non-sleeping sides were determined according to PR SL in those who reported a preferential sleeping side (n=23/28). The five patients who did not report a preferential sleeping side were excluded from the PR SL analysis. The mean value of the UEDA distance on the sleeping side was 4.9 ±1.7 mm, while the corresponding figure for the non-sleeping side was 4.1 ±1.5 mm. A paired t-test analysis showed that the difference in laxity was statistically significant (t=6.340, df=45, p<0.01, two-tailed).

Spearman’s ρ correlation analysis was employed to assess the relationship between PR SL, ID SL and PSG SL. A significant association was found between PR SL and ID SL. The association between PSG SL and either ID SL or PR SL was not found to be significant (Table 2).

**Table 2:** Spearman’s ρ two-tailed correlation analysis between investigator-detected and patient-reported sleep laterality A high correlation was detected between ID SL and PR SL but not for ID SL-PSG SL and PSG SL-PR SL PSG: polysomnography; ID SL: investigator-detected sleeping laterality; PR SL: patient-reported sleeping laterality; PSG SL: polysomnography-detected sleeping laterality

	r_s_	N	P-value
Investigator-detected sleep laterality vs. patient-reported sleep laterality	0.330	56	0.013
Investigator-detected sleep laterality vs. PSG sleep laterality	-0.007	56	0.957
PSG sleep laterality vs. patient-reported sleep laterality	-0.108	56	0.430

## Discussion

While FES was first described by Culbertson and Ostler in 1981, its pathogenesis remains unclear [1,4,5]. The prevalence of FES in the general population is approximately 2.3-3.8% and is higher in patients with OSA.

FES often affects the eyelid that corresponds to the side of the head on which the patient customarily sleeps [4,5]. As a result, the role of repetitive mechanical loading in triggering an adaptive response within the tarsal plate fibroblasts has been proposed. This involves a resetting of the tensional homeostat, causing the tarsal plate to maintain tensional homeostasis only at higher mechanostat set points [6]. There is also evidence suggesting that matrix metalloproteinases (MMPs) are elevated in patients with OSA. MMPs are a group of zinc-containing endoproteases, which, when in excess, may result in the structural degradation of tissue [7]. Further, altered elastic fibre phenotype and collagen accumulation within the tarsal plates of FES patients have previously been reported [8]. Combined, these hypotheses may explain the lid hyperlaxity characteristic of FES.

OSA is a chronic condition caused by a complete or partial obstruction of the upper airway during sleep, causing snoring, choking, frequent nocturnal awakenings, disrupted sleep, and daytime hypersomnolence [9,10]. It is confirmed diagnostically through PSG, which objectively measures the frequency of respiratory disturbance events during sleep [10,11].

In the adult population, the diagnosis of OSA is defined by more than five apnoea and hypopnoea events per hour of sleep associated with daytime hypersomnolence. The prevalence of OSA is approximately 2-5% in women and 3-7% in men [10,11]. The ratio of hypopnoeic episodes and hours of sleep is expressed by the apnoea-hypopnoeas index (AHI). Patients with an AHI of <5 are considered to be normal or simple snorers. Patients with an AHI of ≥5 to <15 are classified as demonstrating mild OSA; those with an AHI of ≥15 to <30 are considered to have moderate OSA; patients with an AHI of ≥30 are classified as cases of severe OSA.

OSA is an independent risk factor for cardiovascular and cerebrovascular disease, and metabolic disorders including type 2 diabetes. These include sudden death, myocardial infarction, stroke, depression, anxiety, hypertension, waking unrefreshed, daytime somnolence, dementia, loud snoring, apnoeic episodes with sudden distressed waking and microsleeps (occasioning sudden death when driving) [10]. In addition, erectile dysfunction [12] and associated disturbance of sleep, not only of the patient but also of his/her sleeping partner [13], are all reported consequences of OSA. Thus, active management of OSA can make a significant contribution to a patient’s health.

OSA is also associated with numerous ophthalmological conditions including FES, associated with painful eyes on waking because of corneal ulceration and papillary conjunctivitis, non-arteritic anterior ischaemic optic neuropathy, progressive astigmatism-against-the-rule, and keratoconus. Upper and lower eyelid ptosis, lower lid entropion and ectropion, and normal-tension glaucoma are also associated with it [3-5,12-14].

This study is the first to examine the use of PSG as an accessory tool in the diagnosis of FES by looking at the relationship between PR SL, ID SL, and PSG SL.

Correlation between PR SL and ID SL

The findings of this prospective, multidisciplinary, masked, single-centre study demonstrated a significant correlation between PR SL and ID SL. Additionally, the findings demonstrated a significant difference in upper eyelid laxity between dominant sleeping and non-dominant sleeping sides. This supports the hypothesis proposed in a study by Figueira et al. [3] (a study that included some of the current authors) that PR SL and ID SL are significantly correlated [1,2,12,15]. The literature has frequently reported the association between FES and PR SL, which was also found to be statistically significant in this study. However, the current study is the first to analyse the association between lid laxity and sleeping laterality using PSG.

Chambe et al. have reported that outright FES is more commonly observed with increasing severity of OSA [4]. While not evaluated by the current study, it is probable that the lax eyelid diagnosed by UEDA could be a precursor to FES. Therefore, the diagnosis of a lax eyelid could possibly be used as an indicator of early OSA. This should prompt the clinician, whether respiratory physician, ophthalmologist, or family medicine practitioner, to attempt to confirm the likelihood of OSA by evaluating eyelid laxity as described above (under Eyelid examination in Materials & Methods).

The application of UEDA is useful in determining sleeping laterality and the possible diagnosis of OSA. Not only is it straightforward to perform, but it is also economical, and takes generally no more than 5-10 seconds of the clinician’s examination time. Adding to the work of Figueira et al., the current study confirms the reproducibility of the LESC study [3]. It could potentially be adopted as a primary method of directing the clinician towards an earlier diagnosis of OSA. It is also appealing because by following the simple UEDA protocol documented above (under Eyelid examination), all physicians may perform it with ease.

Given the sequelae of FES, as outlined above, referral to an ophthalmologist may be warranted for further detailed visual system assessment of the patient with suspected OSA. This is important clinically, as there are effective medical and surgical interventions to manage the eyelids and ocular surface in FES, as well as the ophthalmological complications of lax eyelids in general. Our group recently described what appears to be a highly effective new technique for surgical management of FES [16].

Limitations of PSG in diagnosing sleeping laterality

The current study disclosed a discrepancy in defining sleeping laterality between its different assessment techniques. The finding that PSG SL was not significantly associated with either ID SL, or PR SL, was unexpected, highlighting potential issues in objectively determining sleeping position by PSG, given the current technology. This indicates that PR SL and ID SL are useful in determining sleeping laterality and may facilitate the diagnosis of FES and OSA.

The lack of association between PSG SL and sleeping laterality by PR SL and ID SL suggests that the underlying issue may not be subjective patient-reporting, but rather the reliability of the PSG data using current techniques. Indeed, the inaccuracies of positional sensors used in PSG in determining sleeping positions have been previously reported [17]. It is also pertinent to note that at the DSRM, positional data were obtained through single positional sensors located on the midline of the abdominal wall. Furthermore, discrepancies between head position and torso position, even in studies using dual positional sensors, have also been previously observed [18].

The relatively disruptive nature of a PSG assessment to an individual patient may also play a role in changing patient-sleeping patterns [17,18]. A phenomenon called ‘first-night effect’ describes alterations in sleep architecture due to discomfort caused by PSG machinery and electrodes. The interpretation of a single set of data, as was performed in this study, may also discount the impact of intra-individual variability on sleeping patterns [18]. These confounders may potentially affect the accuracy of the positional data obtained with PSG.

Thus, despite the limitations of PSG in detecting sleep laterality, UEDA appears to have a promising role in the future of OSA diagnosis. Further research is required to determine if UEDA can be utilised as a definitive screening tool for OSA, and larger prospective observational studies may be required. These should consist of improved sleep laboratory technology for detecting head position, particularly in relation to eyelid compression by the pillow.

## Conclusions

Detection of upper eyelid laxity or FES by the ophthalmologist, family medicine practitioner, or respiratory physician may ultimately assist in confirming the diagnosis of OSA. If UEDA is employed as a screening technique, and demonstrates asymmetric and prominent eyelid laxity, OSA may be considered as a possible underlying diagnosis. While increased upper eyelid laxity was confirmed to be associated with sleeping laterality in OSA, PSG SL correlated poorly with ID SL and PR SL. This may be explained in part by technical limitations implicit in current PSG techniques.

In the future, the objective determination of sleeping laterality may be assisted by more sophisticated head-sensing and eyelid position-sensing technology in sleep laboratories. Individual patients with suspected OSA may warrant referral to an ophthalmologist for evaluation of lax eyelids as well as FES and associated ophthalmological abnormalities.
